# Treatment of Frequently Recurring “Erysipelas” of the Face

**Published:** 1882-03

**Authors:** 


### TREATMENT OF FREQUENTLY RECURRING "ERYSIPELAS" OF THE FACE.

Dr. James Braithwaite (*British Medical Journal*, vol. I., 1881, p. 681) says that for many years his father and himself have used with entire success a strong solution of tannin (four to eight grains to the drachm of alcohol and water). This application, which is not disagreeable to the patient, should be painted over the parts affected with a soft brush every two or three hours, and allowed to dry, the patient being careful to keep the face from the fire. If there is a tendency to frequently recurring "erysipelas," it is well to keep the tannin at hand, as it will always arrest a threatened attack.

